# A Review of Miniature Radio Transmitters for Wildlife Tracking

**DOI:** 10.3390/s25020517

**Published:** 2025-01-17

**Authors:** Sivan Toledo

**Affiliations:** The Blavatnik School of Computer Science and AI, Tel Aviv University, Tel Aviv 69978, Israel; stoledo@mail.tau.ac.il

**Keywords:** wildlife tracking, transmitter localization, miniature transmitters, radio transmitters, tracking tags

## Abstract

This article surveys the literature on miniature radio transmitters designed to track free-ranging wild animals using emitter-localization techniques. The articles covers the topics of power sources used in such transmitters, including miniature batteries and energy harvesting, techniques for generating the transmitted radio-frequency carrier, techniques for creating short radio pulses and more general on–off schedules, modulation in modern wildlife-tracking transmitters, construction, manufacturing, and tuning techniques, and recent trends in this area. The article also describes the recreation of the first successful wildlife-tracking transmitter, a nontrivial invention that had a profound impact on wildlife ecology, and explores its behavior.

## 1. Introduction

Movement is a fundamental aspect in the lives of many types of organisms. Animals move to find food, water, and shelter, to evade predation, to find a mate, and to avoid harsh weather [1]. Therefore, tracking free-ranging wild animals is a key technical challenge in ecology. While global navigation satellite systems (GNSs) like the global positioning system (GPS) allow ecologists to effectively track many types of wild animals, many animals require other, more specialized, tracking technologies [2]. This article focuses on one critical component of some of these specialized technologies: miniature radio transmitters (tags) that are attached to wild animals to track their movement. We focus on tags that are localized using the transmitted signal, not tags that use radio transmissions to upload locations estimated using a GNSS receiver (e.g., [3]).

Radio transmitter localization emerged at the early 1960s as an effective way to track wild animals. For decades, attachment of a radio transmitting *tag* was essentially the only way to track many species of wild animals. GPS receivers (and more recently multi-constellation GNSS receivers) replaced radio tags as the preferred tracking method for many species, but radio transmitter-localization remains essential for many other species. This family of techniques still delivers valuable scientific data that cannot be obtained otherwise (see, e.g., [4,5,6,7,8,9,10]). For a review of other significant wildlife tracking systems, see the survey by Nathan et al. [2].

This paper surveys the scientific literature on miniature transmitters for wildlife tracking. We do not cover wildlife-tracking transmitters offered by commercial vendors, mostly because they are either based on published designs (e.g., J. S. Lotimer founded in 1984 a company called Lotek, which commercialized transmitters he developed while in the public sector [11]) or there is not enough information about them. In some cases articles in the literature describe commercial transmitters (e.g., [12]).

How small should a tag be to be considered miniature? The viewpoint in this article is that a tag is considered miniature if designing and manufacturing it requires specialized engineering efforts, efforts that are not required in the absence of mass and size constraints. These specialized efforts often lead to specialized designs. We adopt this viewpoint because over the 65 years that the article covers, from 1959 to 2024, the size of the smallest useful tag has shrunk dramatically. In 1963, a 10 g tag [13] was considered miniature, for good reason: it improved upon a 1959 design that weighed 122 g [14]. Today, 10 g tags are fairly easy to design, but smaller tags are not; tags that weigh 1 g or below certainly require specialized engineering efforts, and sub-0.2 g tags even more so [15,16].

### 1.1. Radio Transmitter-Localization Wildlife Tracking Systems

Radio transmitter-localization tracking systems typically aim to estimate the trajectory of an animal over time, represented by a series of timed point estimates. Ideally, the system also estimates the uncertainty of both the time stamps and the location estimates (but the absolute accuracy of time stamps is often not of much importance). Typically, the transmitter emits periodic transmissions, often coded. Receivers that are part of the localization system detect the transmissions, identify the transmitter (and hence the animal), and measure aspects of the signal that allows the transmitter to be localized. The location of receivers is usually known. The location of the animal is estimated by minimizing the residual of a system of constraints. The residual measures the discrepancy between the measured property of the signal and the prediction of that property given a hypothetical transmitter location *ℓ*.

If a receiver can estimate the direction to the transmitter, the animal can also be localized using a labor intensive technique known as homing in. The receiver is placed at some location $\rho_{1}$ (its coordinates need not be known exactly) from which the azimuth to the transmitter is estimated. The receiver, typically carried by a person on foot or in a vehicle, is moved in that direction to a new location $\rho_{2}$, presumably closer to the transmitter. This is repeated until the animal carrying the transmitter can be visually observed and its location recorded. This often works if the animal is moving during the process, as long as it moves slowly compared to the person performing the procedure.

In some cases, all or a subset of the receivers are carried by wild animals, often of the same species. These receivers detect transmissions by nearby transmitters carried by other individuals, identify the transmitter, and record the time of detection. This serves as a record of the two animals, the one carrying the transmitter and the one carrying the receiver, being in close proximity at that point in time. Such records allow ecologists to build graphs of social networks of groups of animals, which are informative even when the physical locations (the coordinates) where the two animals met are not known. We refer to such systems as encounternets, the name of the first system of this type [17,18,19].

The first transmitter-localization wildlife tracking systems localized animals using direction-of-arrival (DoA) estimates. The DoA estimates were derived from relative-signal-strength measurements (RSSIs). The direction to the transmitter in these early systems was estimated by rotating a directional antenna and searching either for a signal-strength maximum or for a signal-strength minimum. It is easier to build antennas that produce sharp minimums, also called nulls, but both forms are used. For homing in, the signal strength is usually estimated manually, by listing to audio output of a receiver [13,20]. In systems with fixed receivers, the antenna was rotated mechanically and the signal strength was recorded on film [21]. Locations were estimated by triangulation.

Modern DoA wildlife tracking systems tend to rely on comparing the signal strength arriving at several co-located antennas with different radiation patterns, often Yagi antennas oriented in several directions [10,22]. In principle, the direction of arrival can also be estimated from the phase of the signal at different antennas (typically omnidirectional), but I am not aware of wildlife tracking systems using this principle. The direction to an unmodulated transmitter can also be estimated using a single-channel receiver whose input is fed through a switch by four or more omnidirectional antennas. The cyclic switching between antenna frequency modulates the received signal. The application of this technique, called pseudo-Doppler direction finding, for wildlife tracking is being investigated [23,24,25], but it does not appear to have been used in the field yet.

The direction of arrival of a signal can be estimated from signal strength or phase measurements even if the signal has low bandwidth, and indeed many wildlife tags that are used with such systems transmit low-bandwidth on–off-keyed signals (typically around 1 kHz, depending on the shaping of the pulses).

The location of a transmitter can also be estimated from measurements of the Doppler shift of the signal. The ARGOS system consists of receivers carried by weather satellites [26]. The receivers can detect transmissions, identify the tag (and recover other information transmitted, beyond the tag’s identifier), and estimate the Doppler that the signal underwent due to the speed of the moving satellite. This has been used successfully to track birds and marine species (when they surface) [27,28,29].

Time-of-arrival (ToA) localization, which was pioneered by GPS, is now also used in so-called reverse-GPS systems to localize transmitters attached to wild animals [5,15,30,31]. In general, these systems consist of a group of receivers with omnidirectional antennas. The receivers identify transmissions from tags, estimate their arrival times, and report to a server that estimates the location of the transmitter from reported arrival times.

### 1.2. Requirements and Desirables for Miniature Transmitters

The utility and applicability of a wildlife tracker that contains a radio transmitter depends on a few characteristics, which we list in the following paragraphs. Our focus is primarily on technical characteristics and on the viewpoint of tag designers. The same characteristics are also significant to users, but their viewpoint and understanding of these properties is naturally a little different. Table 1 lists properties of tags that are relevant to users and explains their relevance. The key technical characteristics of tags are as follows:

**Low Mass and Small Size**
  The distribution of animal species as a function of body mass is skewed; there are many more species with small body mass than species with large body mass. Therefore, a miniature tag is applicable to more species than a physically large tag. The challenge of making tags smaller and lighter is a constant feature of this field.
**Efficient Omnidirectional Antenna**
  Ideally, the antenna should convert all the power delivered to it into electromagnetic energy. In addition, the radiation pattern should be omnidirectional or hemispherical to ensure that the antenna radiates in the direction of receivers. Antennas with hemispherical radiation patterns, directed away from the animal’s body, minimize radiation absorbed by the animal’s body. The desire that the antenna radiates most of the delivered power often conflicts with the requirement that the tag be physically small.
**Wildlife-Friendly Antenna**
  Some antenna topologies, like loops, can be dangerous to animals because they can entangle with vegetation or other structures. Whip antennas are often acceptable and preferred.
**Energy-Harvesting or Primary Batteries**
  The energy required to power the tag must either be carried in batteries or generated by energy-harvesting devices, such as solar panels. While energy-harvesting devices are desirable, especially for long-lasting tags, they sometimes limit miniaturization, and they are not effective on some species (e.g., solar panels are useful for most bat species). When energy harvesting is not possible, which is a common case, tags are usually powered by primary batteries.
**Tag Identification**
  In most cases, multiple animals are tagged simultaneously, so the identity of the transmitter must be identifiable from the tag’s transmission. In some cases, tags transmit data packets that contain the tag’s identifier and that can be decoded by the receiver. However, simple tags that transmit continuous-wave (CW) pings are identified by the transmission frequency or an on–off-keying (OOK) pattern or by the pinging rate.
**Frequency Accuracy and Stability**
  Frequency accuracy and stability are crucial for ARGOS tags that are localized by Doppler shifts caused by the receiving satellite’s movement [28]. These properties are also important for tags identified by the transmission frequency. In general, even when the exact carrier frequency is not significant, frequency drift requires more effort in the receiver side, to search for the transmission. This is more significant for narrowband transmissions or wideband transmissions designed for coherent detection [32,33] than for incoherent wideband tags [34,35].
**High Bandwidth**
  The accuracy of time-of-arrival localization depends on the bandwidth of the transmitted signal. Therefore, tags for ToA localization systems must transmit wideband, high bit rate signals.
**Activation Mechanisms**
  Tags with no energy harvesting mechanisms are usually encapsulated together with their batteries to protect the electronics from the environment, well before they are deployed. In miniature tags with small batteries, the ability to switch the tags between active (transmitting) mode and off or standby mode (no transmissions) is highly desirable.
**Sensors**
  Tags with additional sensors can provide a wealth of information about the physiology, behavior, and environment of an animal. For example, accelerometers, perhaps the most useful and ubiquitous sensors, provide insight into the animal’s behavior [36]. Barometric altimeters can provide more accurate altitude estimates than most terrestrial transmitter localization systems [35,37]. On-board sensors require either a large memory, usually nonvolatile, to store measurements, and/or a capability to upload sensor data to base stations via radio links. Sensors can also modify the transmission schedule of radio tags. For example, sensors can be used to transmit only when an aquatic animal surfaces [38] or they can modify the transmission schedule upon mortality [11,39]. Sensors and on-board storage of sensor data are mostly orthogonal to issues pertaining to radio transmissions and are therefore not covered further in this article.

**Figure 1:**
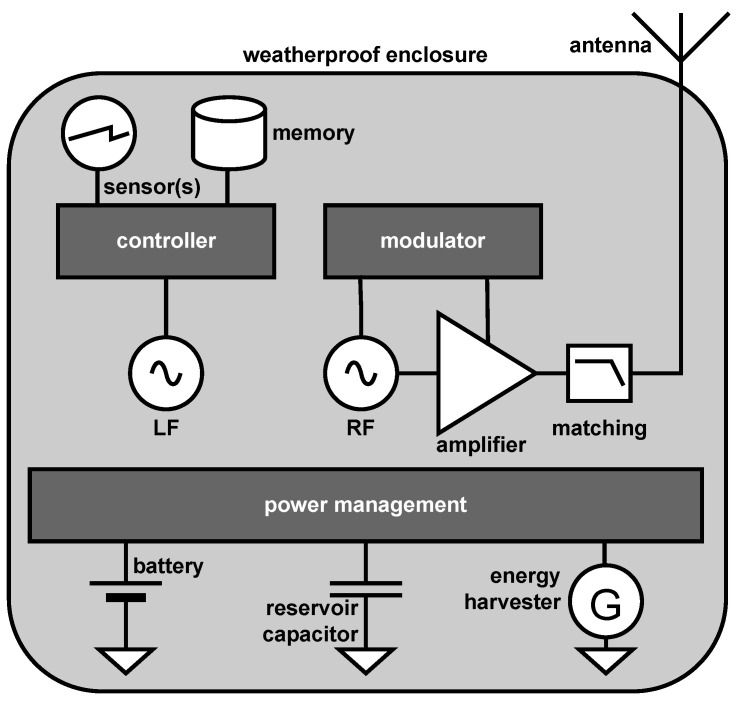
The components of a radio-transmitting tracking tag. All of the components except for the RF generator, the matching circuit, and the antenna are optional, but almost all tags also include a low-frequency (LF) oscillator, a controller, and a modulator. The function of the different components and typical designs for them are described throughout the article.

The rest of this article surveys the literature on miniature radio transmitters for wildlife tracking, focusing on their key building blocks, shown in the diagram in Figure 1. Section 2 describes power management and power sources for such tags. Section 3 describes the circuits that generate the radio frequency (RF) signal that the tag emits. Section 4 describes circuits that turn transmissions on and off, primarily to conserve power and spectrum utilization, but sometimes also to identify the tag or code information. Section 5 describes more sophisticated modulation schemes, mostly for use in time-of-arrival localization systems. Section 6 covers antennas. Section 7 describes construction, manufacturing, and tuning methods. Section 8 describes recent trends in tag design. Section 9 describes particularly significant examples of deployments of transmitting tags described earlier in the article. Section 10 concludes the article with a high-level discussion of both the history, the state of the art, and the foreseeable future in this area.

## 2. Power Management and Power Sources

Wildlife tracking tags (of all types) are powered by batteries and/or energy-harvesting devices (mostly solar cells). Efficient use of energy is critical for maximizing the life spans and the utility of tags, especially extremely light-weight ones. More specifically, most transmitting tags are powered by primary batteries. In such tags, minimizing energy losses extends the life spans of the tags. Energy efficiency is also important in tags with tiny energy harvesters; the small size reduces weight but also the average power available.

We begin this section with a description of the types of batteries and energy harvesters that are used in miniature transmitting tags and then describe power and energy management principles and techniques.

### 2.1. Batteries and Energy Harvesters

Most miniature transmitters designed for wildlife tracking are powered by primary batteries. The most common primary miniature batteries today are lithium manganese (lithium coin cells, such as CR1025, CR2032, etc.), lithium thionyl chloride (larger, usually cylindrical), silver oxide (e.g., type 337 at 0.17 g and larger types), and zinc air, which are mostly designed for hearing aids but have been used in tracking tags [17,40]. In the past, mercury batteries were widely used in tracking tags [13], but they are now obsolete due to their toxicity.

Zinc–air batteries tend to have the best energy density, since one of the chemicals that they use, air, is taken from the environment, not stored in the battery; but they are often more difficult to use successfully.

Most of the batteries that are used in wildlife tracking tags are designed and produced for other markets, like hearing aids, remote control, and watches (the same is true for most of the other components in wildlife tags). However, there have also been efforts to design and manufacture batteries that are optimized for wildlife tags [41]. Chen et al. designed such a battery for ultrasonic wildlife tracking tags tags for fish. It was later also used in radio tracking tags [16]. This 6 mAh 0.065 g lithium carbon flouride battery has relatively high voltage (above 3v), high energy density, long shelf life, and wide operating temperature range. This battery is currently used in two commercially available tags, an underwater ultrasonic tag [42] and a radio tag [43].

Batteries convert chemical energy to electrical energy. The amount of power that they can deliver depends on the rate at which the chemical reactions that perform the conversion can happen. That rate is limited by the physical size and physical structure of the battery (e.g., the size of its electrodes). Most miniature batteries cannot deliver high power. This is often modeled as internal resistance.

Transient changes in the distribution of chemicals in a battery can further reduce its ability to deliver power. Abstractly, its internal resistance rises. Diffusion of chemicals inside the battery resolves this eventually, but slowly. This phenomenon is called polarization concentration [44]. Its likelihood increases if significant power is drawn from the battery, even if the consumer is pulsed.

The main type of energy harvester used in wildlife tracking tags are small solar panels [12,45]. On fairly large tags (designed for boars and similar animals), kinetic energy harvesters have also been used [45]. The energy conversion rate of energy harvesters is also limited, obviously. In some harvesters, in particular solar panels, the conversion rate depends on the impedance presented to the harvester.

Today, the power delivery characteristics of energy harvesting chips are matched to the characteristics of energy storage devices (rechargeable batteries and capacitor) using specialized energy-harvesting chips. In particular, transmitting tags have dedicated nano-power energy-harvesting chips such as bq25570 or bq25504 [12], or ADP5091 [45].

### 2.2. Energy and Power Management

Energy management techniques used in wildlife tracking tags (and other battery-operated and energy-harvesting devices) can be partitioned into three categories: duty cycling, impedance matching, and avoiding losses.

Virtually all wildlife tracking tags use duty cycling, switching between short high-power activity periods and long deep sleep periods in which they use very little energy. Ever since wildlife radio tracking was invented, transmitting tags have been transmitting only during activity periods [13,14]. These transmissions are often called pings in the literature. Other types of tags sense and process sensor data during activity periods, or communicate with infrastructure base stations or with other tags [10,17,18,35]. Pinging mechanisms are covered in more detail below, in Section 4. Duty cycling is usually a trade-off. In a transmitting tag, pinging more often can potentially generate more localization, improving the temporal resolution of the track (and through smoothing, also its spatial resolution). Longer pings improve the signal-to-noise ratio at the receiver, which often results in more accurate localizations. But longer and more frequent pings increase the average power consumed. With primary batteries, the increase in average power shorten the tag’s life span. With energy harvesting, the average available power is limited by the harvester.

Duty cycling sometimes involves additional, more subtle trade-offs. For example, in tags with sensors, such as accelerometers or air-pressure sensors, the sensor can often be configured to deliver averages of raw samples (oversampling). This improves the accuracy and resolution of sensor measurements, but it also extends the duration of activity periods or draws more power, thereby consuming more energy per measurement.

Tags have one or two power sources, a battery and/or harvester, and one dominant power consumer, the transmitter. In many cases, the power sources cannot produce enough instantaneous power for the transmitter. In addition, the power delivery characteristics of energy harvesters is often ill-matched to that of rechargeable batteries or reservoir capacitors (e.g., they do not produce the appropriate voltage). Therefore, tags often contain devices designed to match impedances or power deliver/consumption rates.

The most common matching device in tags with miniature batteries is a so-called reservoir capacitor [10,44,46]. In some cases, the primary or rechargeable battery of the tag cannot provide enough power to power the transmitter, or it can do so but at the risk of transient failure of the battery. To address that, the battery is used to charge the reservoir capacitor. Capacitors have very low impedance, so they can easily deliver high power to the transmitter. The delivery of power depletes the charge on the capacitor (faster than the battery can replenish it) and the voltage across it drops; the transmitter must be able to cope with this characteristic.

The low impedance of reservoir capacitor exacerbates polarization concentration [44]. To mitigate that to some extent, the reservoir capacitor can be charged through a current-limiting resistor.

Even with a current-limiting resistor, polarization concentration can still occur [44]. Some tags mitigate such failures by detecting the them (by measuring the voltage on the capacitor) and delaying the next transmission until the battery recovers [35]. Obviously, this mitigation mechanism should consume very little power, otherwise the mechanism itself fails.

Nano-power energy harvesting chips perform several functions. Most importantly, they raise or lower the voltage delivered by the harvester to a level that is appropriate for charging an energy-storage device, a battery or capacitor. Many of them also include a mechanism called maximum power point tracking (MPPT), which senses periodically the power delivered at different impedances and presents to the harvester a nearly optimal impedance. These devices can also regulate voltage to the load (the transmitter), let the load know if there is enough power stored or not, etc.

Finally, effective tag designs avoid energy losses, which can occur due to a variety of reasons. A current-limiting resistor wastes power; by sizing it carefully, the design can balance between this energy loss and the risk of polarization concentration. Voltage regulators waste power; linear regulators waste more than switching ones. Early tags avoided regulators entirely, eliminating both the energy waste and the bulk (in some types of batteries, this causes transmissions to weaken as the battery is depleted). Sophisticated tags require voltage regulation; many tags today are driven by chips that contain both linear and switching regulators; choosing to use a switching regulator minimizes energy loss, often at the cost of additional components (inductors) and therefore more bulk [35]. High-capacitance physically small reservoir capacitors are leaky [44,46]; this leakage also wastes energy. This presents tag designers with yet another trade-off: they can use a tiny battery and a tiny leaky capacitor or a tiny battery with a larger capacitor that leaks less or a large battery without a reservoir capacitor.

Leaky reservoir capacitors are just one example of potential energy loss that occurs even between activity periods (pings). Many electronic circuits draw some power even when they are not doing anything useful. Because tags spend almost all their time between activity periods, minimizing this energy loss is critical. Early tags address this concern using appropriate analog circuit technique, such as the use of class-C amplifiers [20]. Modern tags are driven by low-power microcontrollers which disconnect most subsystems from power when the microcontroller enters a deep sleep mode. When the tag includes devices other than the microcontroller, they are usually turned off or disconnected from power programmatically [16].

One kind of energy loss that is somewhat unique to wildlife tracking tags is operation before the tag is actually deployed on an animal. Due to physical size to waterproofing, mechanical on–off switches are not a viable option. Avoiding transmission until the tag is actually deployed extends the useful life span of the tag and eliminates useless spectrum usage. In the past, magnetically operated switches called reed switches were popular [20], or even soldering together power delivery wires in the field (and waterproofing the connection). Today, transmission is often turned on and off using magnetic [35] or light [16] sensors.

## 3. Radio-Frequency Signal Generation

The first challenge in a miniature radio transmitting tracking tag is to generate the radio-frequency signal. This section covers this aspect.

### 3.1. Single-Stage Power Crystal Oscillator

**Figure 2:**
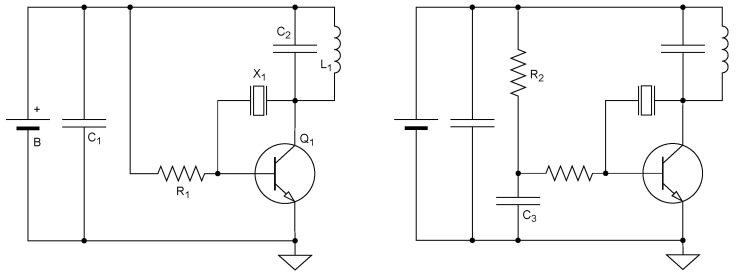
Single-stage power-oscillator tags. The circuit on the left is of the Cochran–Lord CW tag [13,47] and the one on the right is of the Tester–Warner–Cochran pinging tag [48]. The original circuits used PNP transistors, not NPN ones.

In the early 1960s, William Cochran and his colleagues at the Minnesota Museum of Natural History developed highly effective and highly influential single-stage wildlife tracking transmitters [13,47,48]. In a seminal article, Cochran and Lord described the basic RF generating circuit, shown in Figure 2 (left). The circuit is a minimalist crystal oscillator. It consists of a transistor, $Q_{1}$, a current-limiting base resistor $R_{1}$, a quartz crystal $X_{1}$, and a resonant tank circuit consisting of $C_{2}$ and $L_{1}$. The original circuit used a (germanium) PNP transistor; we draw the circuit with an NPN transistor, which became the norm later. In Cochran and Lord’s tag, $L_{1}$ was a single-turn inductor that also served as the antenna. It encircled the neck of the animal and was part of the collar that also carried the tag.

In the wildlife-tracking literature, this type of circuit is usually referred to as a single-stage tag, to emphasize the lack of a buffer or power amplifier stage. In the electronic circuits literature, this circuit is sometimes referred to as a power oscillator, to emphasize the fact that the same stage both generates the RF signal and delivers significant power to the load.

The power supply for the transmitter consists of one or more batteries, denoted *B*, and a decoupling capacitor $C_{1}$. Originally, 1.4 V mercury batteries were used, but the circuit works fine with a wide range of batteries, including 1.5 V silver oxide batteries (the smallest widely available cells today), 3 V lithium-manganese coin cells, and many more.

The Cochran–Lord circuit transmits continuously, which is not necessary for wildlife tracking. It was quickly augmented to periodically transmit short pulses by adding resistor $R_{2}$ and capacitor $C_{3}$, as shown in Figure 2 (right). We explore this modification in Section 4 below. This basic circuit has been used for decades, with variations in both the pulse-generation mechanism (here $R_{2}$ and $C_{3}$) and in the tank circuit.

**Figure 3:**
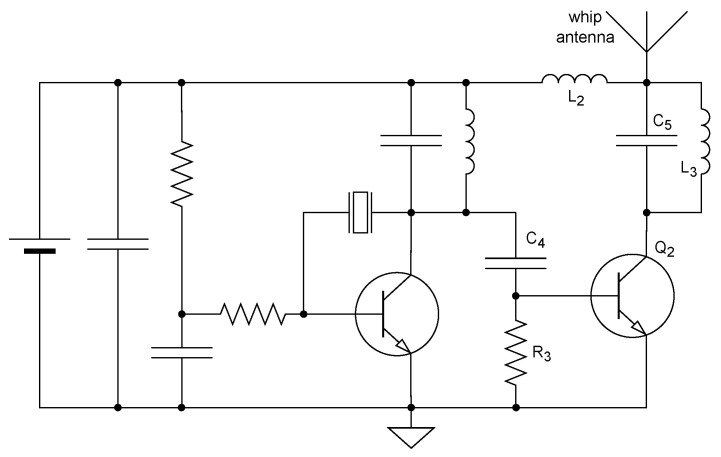
A two-stage tag, from [20].

The variations in the tank circuit are designed mainly to improve the spectral purity and hence the efficiency of the tag. The early 1960s tags [13,47,48] operated at the frequency of the crystal, around 26.5 MHz. These early articles did not report on harmonic suppression, but there really was not any, and it is likely that much of the power was radiated at harmonics of this frequency. The harmonics both reduce the power that is delivered to the receiver, which is tuned to the fundamental frequency, and possibly cause interference to other users of the electromagnetic spectrum. Single-stage tags designed from the late 1960s mostly operated at harmonics of the crystal’s fundamental frequency, usually around 140–160 MHz, and used more elaborate tank circuits designed to concentrate as much as possible of the power at a single frequency. For example, the circuit described by Kenward [20] (who attributes it to a 1967 Cochran design) uses a tank with two inductors and two capacitors, Naef-Daenzer uses two and one [40] (as in Figure 3), and Naef-Daenzer et al. use three and three [49].

The transition to very high frequencies (VHFs) also enabled the use of whip antennas, which are much more appropriate for many types of animals than loop antennas. The tank circuits also help in antenna matching, although these whips are often short relative to the wavelength and fairly inefficient.

Tag masses for such tags started at 10 g excluding the battery in the early 1960s [13], dropped down to 1.2 g in the 1980s [20], to 0.4 g in the 1990s [40], to 0.2 g in the 2000s [49], and to 0.11 g in the 2010s [30].

### 3.2. Two-Stage Oscillator–Amplifier Transmitters

One of the disadvantages of the single-stage tag design is that changes in the impedance of the load (the antenna) change the oscillation frequency. Such changes can easily be caused by movement of the antenna relative to the animal’s body or by the antenna becoming close to other animals or the the ground [13,47]. This was already noted by LeMunyan et al., whose efforts to locate woodchucks using two-stage low/medium-frequency tags predate the Cochran–Lord design [14].

Adding a second stage, as in the tag circuit shown in Figure 3 [20], isolates the oscillator from the load to improve frequency stability, and can also increase power output. In this circuit, $Q_{2}$ serves as a class-C power amplifier, with a tank circuit consisting of $C_{5}$, $L_{2}$, and $L_{3}$ driving a whip antenna. Similar tank configurations are also widely used in single-stage tags [40].

The design of the second stage in Figure 3 is also minimalist, helping to keep the component count and hence the mass down. Still, the second stage adds bulk and in low-power tags also reduces the energy efficiency of the tag. Two-stage tags designed under laxer weight constraints can use a variety of circuit topologies to optimize frequency stability, power consumption, and spectral purity. For example, the two-stage tag described by Broekhuizen et al. [38] uses a carefully biased oscillator (using four resistors whereas the minimal design uses one) and a grounded-base amplifier.

In some two-stage tags, the second stage acts as a frequency doubler. For example, Kolz et al. [39] describe a tag in which the first stage oscillates at the third harmonic of a 27 MHz crystal, and the second stage acts as a doubler, emitting a 162 MHz signal. The tag described by Broekhuizen et al. [38] also uses frequency doubling. Frequency multiplication by amplifying harmonics was also used on more complex tags. For example, Koltz et al. [50] describe a 401.2 MHz transmitter for satellite Doppler tracking, in which the ultra-high-frequency (UHF) signal was generated by multiplying the signal from a temperature-compensated crystal oscillator (TCXO).

### 3.3. Phase-Locked Loops

Modern tags generate the radio frequency using a voltage-controlled oscillator (VCO) that is frequency and phase locked to a crystal oscillator operating at a much lower frequency. This architecture, commonly known as a phase-locked loop (PLL) synthesizer, offers frequency agility and does not require any frequency-specific component (the quartz crystal).

This architecture, while ubiquitous, is complex, so it became widely used in wildlife tags only when electronics became integrated enough to implement the entire synthesizer, sometimes with additional functions like a power amplifier and/or a microcontroller, on a single silicon chip. However, some wildlife tracking tags used PLLs that were not completely integrated. For example, Strikwerda et al. [28] describe a 401.65 MHz transmitter designed to be tracked by the ARGOS satellite system. It uses a VCO whose output is divided by 80 and locked to a TCXO. The 170 g tag consists of four printed circuit boards carrying around 20 integrated circuits (some of the complexity was due to other functionality, but the synthesizer was not fully integrated).

Later tags used integrated synthesizers (e.g., to produce a 140 MHz signal [15]), transmitter chips that integrate a synthesizer, a modulator, and a power amplifier [32], transceiver chips [17,19,34], and radio-frequency microcontrollers (RF MCUs) that integrate a microcontroller with a transceiver [10,35].

In some cases, avoiding extreme integration, such as found in RF MCUs, can lead to smaller tags. For example, Lu et al. [16] describe a recent and particularly lightweight tag (0.15–0.16 g) that uses a PLL-based factory-programmable clock module along with a voltage regulator and an MCU. The tag transmits at 164–166 MHz. The clock module is tiny (2 by 2.5 mm) and contains the crystal. Interestingly, Lu et al. propose to program the tag to 1/5 or 1/3 the operating frequency, even though the clock can reach 166 MHz, to reduce the clock’s power consumption (but also the power emitted at 164–166 MHz). This tag is currently available commercially [43].

## 4. Pinging or On-Off Keying

### 4.1. Using a Minimalist RC Network

The simplest way to cause the Cochran–Lord-type oscillators to ping periodically is by driving the base resistor using a resistor–capacitor network, as shown in Figure 2 (right). The earliest reference I could find for this circuit idea in the wildlife tracking literature is from 1962 [51]; Tester et al. [48] describe its use in the simple single-stage tag shown in Figure 2. A relatively large-valued capacitor $C_{3}$ is charged through a large-valued resistor, $R_{2}$. Typical values are 1 μF or more and several hundred kilo Ohms or more. When the voltage on $C_{3}$ becomes high enough for the base emitter diode to conduct, the transistor turns on and oscillation starts. This continues until $C_{3}$ discharges. The first article that showed this subcircuit also included a second resistor, in parallel with $C_{3}$, probably to speed up discharging; later designs omitted this resistor.

The values of $R_{2}$ and $C_{3}$ determine the charging rate and hence the pulse interval. The values of and $C_{3}$ and $R_{1}$ determine the discharge rate and hence the pulse length. However, it is impossible to control the two durations separately with this simple circuit. This is one of the disadvantages of the circuit.

**Figure 4:**
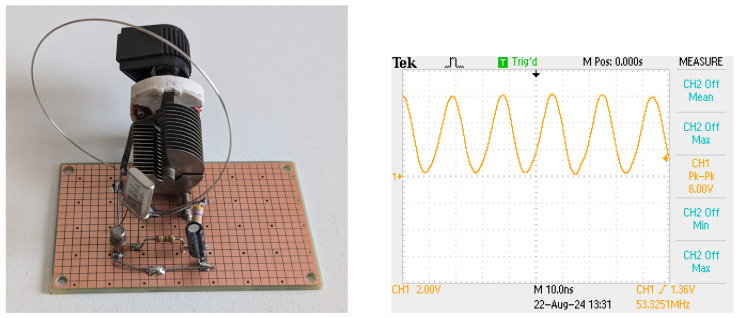
**Left**: A prototype single-stage transmitter. **Right**: Emitter voltage when the tank is tuned and the supply voltage is 3 V.

**Figure 5:**
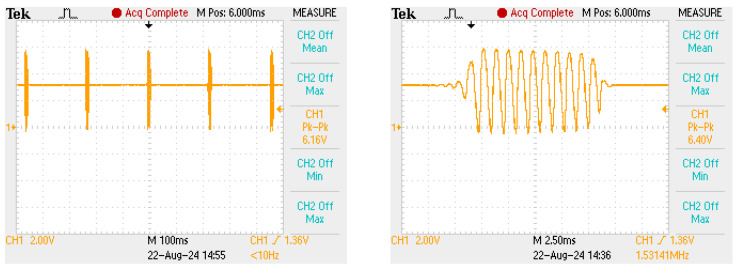
**Left**: Collector voltage in the prototype tag, showing a ping rate of a little less than 10 Hz. **Right**: Collector voltage during one ping.

To experience and to more fully understand this circuit, I built a prototype shown in Figure 4. I started with the circuit of Figure 2 (left). The crystal was marked 32 MHz and seems to be a third-overtone crystal. The transistor is a 2N2222 and the base resistor is 3.9 kΩ. I used a variable capacitor for $C_{2}$ to be able to tune the circuit and a single loop inductor for $L_{1}$. Turning $C_{2}$ produced stable oscillation at about 53.3 MHz, the fifth overtone of the crystal, at voltages ranging from below 1.5 V to 3 V and higher. Adding a 1 μF capacitor $C_{2}$ and a 560 kΩ resistor for $R_{2}$ caused the circuit to ping at around 10 Hz and with pulses of about 10 ms, as shown in Figure 5.

A small improvement on this pulsing mechanism is proposed by Naef-Daenzer et al. [49]. They show that by adding a PNP transistor in parallel with $R_{1}$, the pulse width can be shortened without affecting the pinging frequency.

Note that the same RC pulsing mechanism also works well in the two-stage design shown in Figure 3 due to the use of a class-C amplifier. When the oscillator is shut down, so is the amplifier stage.

### 4.2. Using a Separate Pulse Controller

When the extreme minimalizm of the RC pulsing mechanism is not required, tags use a more modular design with a separate subcircuit that generates pulses that turn on the RF section. This allows controlling the pinging frequency and the pulse width separately and easily.

The introduction of the 4000 series of complementary metal-oxide-semiconductor (CMOS) logic chips by RCA in 1968 enabled a range of designs based on them. Typical designs are described by Skutt et al. [52] and by Kolz et al. [39]. In these designs, two NOR gates form an astable multivibrator, an oscillator with a square-wave output. A monostable multivibrator, consisting of two additional NOR gates, converts each rising edge of the oscillator’s output into a short positive pulse, which in turns feeds the base resistor of the RF oscillator. Anderka [53] achieved the same goals using a CD4047, an integrated multivibrator chip from the same series.

Similar techniques have been used to achieve more complex pulse schedules. For example, Broekhuizen et al. [38] used 4000-series CMOS chips in a tag designed for seals. The tag senses when it is submerged (the salt water creates a conducting path between two metal components), in which case it does not transmit. When the seal surfaces, the tag sends five pings at a rate of 5 Hz and then switches to pining at 1 Hz. Lotimer [11] showed how to use CMOS NAND gates to transmit bursts of pings while controlling the inter-burst interval, the duration of each burst, the interval between pings in a burst, and the duration of each ping. These forms of pulse or on-off-keying modulation allow identification of individual tags (animals) that transmit on the same frequency.

The duration of pings and the length of inter-ping intervals are controlled in these designs by resistors and capacitors. By using temperature- or light-sensitive resistors and motion-sensitive switches, designers could encode information about the animal or its environment in the ping schedule. This was used to sense the animal temperature, level of activity, mortality, and to modify the ping rate between day and night [11,39].

The 4000-series CMOS integrated circuits are specified for operation at 3 V and up. Until recently, there were no alternatives that could operate with a single mercury, silver oxide, or zinc air cell. To control the ping rate and pulse duration in tags that used such cells, discrete three-transistor multivibrators circuits were used [54,55]. Today, there are low-voltage logic families that could be used to implement the logic-gates-based designs, like the SN74AUP that operates down to 0.8 V.

In modern tags, a microcontroller controls the pinging schedule. In many tags, a 32 kHz crystal allows very precise control of the transmission schedule [10,34,35]. When weight must be reduced to a minimum, this crystal is omitted [16], and the schedule is controlled by a low-power low-frequency RC oscillator that is part of the microcontroller. These oscillators are inaccurate. In some cases an inaccurate inter-ping interval is acceptable. In others, a high-frequency crystal oscillator is used to occasionally calibrate the RC oscillator, thus achieving accurate inter-ping intervals.

## 5. Code Modulation for Time-of-Arrival Tags

Time-of-arrival emitter localization benefits from transmitting high-bandwidth long codes, so the codes (the bit sequence that is transmitted) are invariably generated by a microcontroller [15,33,34,35]. The codes are detected and their arrival time estimated by cross-correlation with codes stored in the receiver, so different codes should have low cross-correlation, even at shifts (when they are not aligned), and codes should have low auto-correlation (cross-correlation with the same code, shifted). Some tags use Gold codes, which are specifically designed for this correlation properties [15] but other tags use pseudorandom codes [34,35]. When the codes are long enough, the difference is not significant.

Binary phase-shift modulation (BPSK) is excellent for time-of-arrival estimates, since it allows receivers to perform complex correlation of phase trajectories [32]. This is the modulation used by GPS (newer GNSS use binary offset carrier modulation, which is specifically optimized for time-of-arrival estimation). One tag design relies on an integrated transmitter capable of high-data-rate BPSK modulation, up to 2 Mb/s [32,35]. An older design uses a discrete modulator [15,30].

Another approach is to transmit the code using OOK but to detect it using a BPSK replica at the receiver [33]. In this design, too, the modulator is discrete [33]. This approach simplifies the tag, but it degrades the signal-to-noise ratio (SNR) and hence both the sensitivity of the receiver and the accuracy of the time-of-arrival estimate, since only half the power is emitted.

In general, the use of a discrete BPSK or OOK modulator does not allow controlling the bandwidth of the transmitted signal, so it is unlikely that it would be possible to use this approach to design tags that comply with modern RF emission regulations.

Unfortunately, most integrated RF transmitters/transceivers and most RF microcontrollers cannot emit high data-rate BPSK signals, so most current time-of-arrival tags use frequency modulation (FSK) [34,35]. Unfortunately, the use of FSK degrades both the resilience to interference from other tags and the time-of-arrival estimation accuracy [32].

## 6. Antennas

The vast majority of wildlife-tracking transmitters use monopole antennas: a single conductor that extends away from the tag. The monopole topology minimizes both the interference to the animal and the risk of entanglement. These antennas are often fairly inefficient, radiating only a fraction of the power delivered to them, because their length is around a quarter wavelength or shorter and the tag does not constitute a large enough ground plane Miron [56], Zavrel [57]. For example, Riecken and Raths used 5 cm antennas in 150 MHz tags (200 cm wavelength); see Riecken and Raths [58]. Wikelski et al used 4.2 cm antennas in 378 MHz tags (79 cm wavelength). The need to keep monopole antennas short is well supported by multiple studies: Dougill et al. [59], Jones et al. [60].

Early tags used a wider range of antenna topologies. Perhaps the most significant were loop antennas, which were often part of a collar to which the tag was attached; see Cochran and Lord [13].

Antenna tuning and characterization and the design of antenna matching networks remains a major challenge, especially since most tags use electrically short antennas (short relative to the wavelength), and in particular end-fed monopoles without ground planes or radials. Although vector network analyzers (VNAs), the equipment that is used to characterize antennas, are now affordable [61,62], it is difficult to eliminate the effects of the cable connecting the VNAs to the tag’s antenna (or or a mock-up tag) on the measurements. Although there has been some progress in this area [63], it is still challenging. One technique that has proved useful is to test tag antennas using bottles of a solution prepared specifically to emulate the electromagnetic properties of biological tissue (e.g., a living animal) [64]. Kildal et al. described a laboratory measurement technique that can characterize the properties of UHF antennas in close proximity to biological tissue [65]. Hartsgrove et al. showed how to prepare a solution that emulates the electromagnetic properties of biological tissue (e.g., a living animal) [64], as required in the technique by Kildal et al., as well as in far-field antenna characterization experiments.

## 7. Methods for Construction, Tuning, and Manufacturing

Early miniature tags were constructed by hand using electronic construction techniques that were very different from the established state of the art at the time, which would have resulted in bulky heavy tags. Instead, components were soldered to each other directly without a circuit board or terminal strip [13]. Authors often provided very detailed instructions on how to replicate tags (in some sense, this style of writing mirrors current open-source and open-hardware practices). This continued at least until the 1980s [20] and in some cases into the 2000s [49]. Kenward’s 1987 book offers very detailed instructions on how to construct VHF pinging tags.

The prevalence of surface-mount devices and their increasing miniaturization caused construction methods to shift towards more standard industrial manufacturing techniques using printed circuit boards (PCBs) and automated reflow soldering [10,12,16,34,35] and at least in one case also using injection molding [16].

Even today, industrial manufacturing is used to produce only the tag’s PCB. The process of evolving from a PCB to a complete tag still often requires manual labor, specialized or unusual materials and techniques, and specialized know-how. In particular, batteries are often soldered by hand using specialized technques [66], antennas are often made from unusual materials (steel fishing lines [66], beading wires, and bicycle shifter cables), and many tags are potted in various epoxy materials [67,68,69,70], sometimes mixed with glass bubbles [71] to reduce weight (see, e.g., [16,35]).

## 8. Recent Trends

Several authors recently suggested using Bluetooth low-energy (BLE) tags for wildlife tracking. Farine et al. [72] used commercially available tags (not designed specifically for wildlife tracking) capable of transmitting BLE advertising beacon packets to track wild birds in an urban setting. They programmed the tags to emit messages compatible with Apple’s “Find Me” network. Apple smartphones that receive such packets report them to an Apple server along with the phone’s location, encrypted using an encryption key that the tag sends. The owner of the tag can retrieve these reports and decrypt the locations of the receiving phone, thereby localizing the tag to the vicinity of the reported location. It is not clear whether this network will be open to third-party tags (ones not produced by Apple itself, like its own AirTags), but for now this does work.

Other researchers tagged animals with genuine Apple AirTags, which emit not only BLE packets but also high-bandwidth packets that are better optimized for localization and that can be processed by some Apple phones [73]. These tags are not specifically optimized for wildlife tracking. Custom BLE wildlife tracking tags are starting to appear commercially [74,75], as well as BLE receivers designed to detect them.

Efforts to develop and test tags designed to emit transmissions that are received by commercial radio networks designed for low-volume Internet-of-Things traffic are also under way. For example, Wild et al. [45] tagged animals with transmitters designed for the so-called SigFox network, operating at 800–900 MHz bands. These tags transmit very long messages (6.3 s) very infrequently (a few times a day), yielding limited temporal resolution. The network can localize the transmissions, but the spatial resolution is also coarse (median accuracy of more than 12 km). Some of the tags are very lightweight; some use energy harvesting. It appears that commercial tags that use low-bandwidth cellular standards, specifically LTE-M, may be offered soon [76]. Most tags in this family rely on a GNSS receiver for localization and only use the cellular network to upload localizations, but it appears that one particularly small model is localized by the cellular network. Wild’s dissertation surveys other current wireless standards that might be suitable for wildlife tracking tags Wild [77].

The use of low-Earth-orbit (LEO) satellites to localize a radio transmitter attached to a wild animal is also being explored [78,79]. However, these studies currently focus on assessing the feasibility of this approach rather than on the design of tags. Due to the distance of the satellites from the tag, it is likely that these tags will also require long infrequent transmissions.

In general, in tags that transmit long infrequent transmissions, the additional energy required for GNSS localization is not likely to dominate the energy budget, so in practice the localization in most of these tags (LEO satellite tags, SigFox tags, perhaps cellular tags) might rely on GNSS, with radio transmissions that are used only to upload GNSS localizations, not to localize the tags.

## 9. Examples of Deployments and Impact

To provide a more concrete idea of how miniature transmitting tags are used and of the scientific questions that they help resolve, we describe in this section a selection of wildlife-tracking research projects that used such tags. We focus on representative high-impact examples using a variety of different tags. The list is far from exhaustive; miniature transmitting tags have been used in countless research and conservation projects.

Kenward used single-stage and two-stage transmitters, most likely built by himself [20], to track four goshawks (*Astur gentilis*, although his article uses the former Latin name, *Accipiter gentilis*). The birds were tracked for up to 29 days and the results were used to relate their hunting behavior to the availability of food [80].

Naef-Daenzer et al. tracked fledglings of small songbirds, great and coal tits (*Parus major* and *Parus ater*) using single-stage 0.48 g tags designed by Naef-Daenzer [40] and most likely built by him. The tags used zinc–air batteries and lasted 20–30 days. This project tracked a remarkable number of birds, 221, over a coarse of three years [81].

The same type of transmitters, as well as some commercial transmitters, were used by Bontadina et al. to track 12 esser horseshoe bats (*Rhinolophus hipposideros*) [82]. Bats of this species are tiny, weighing only 4–8 g. The tags weighed 0.33–0.44 g. The study revealed where these bats forage; this was not previously known due to their small size, which precluded tracking them until that point.

Cochran et al. tracked songbirds (*Catharus* thrushes) to demonstrate that they recalibrate their magnetic compass every day [83]. They used commercial 0.7–1 g tags, which were not considered extremely small at the time. However, the discovery was particularly interesting and deep and was published in *Science*.

Knight et al. used tiny tags, close to the engineering limit at the time, to track butterflies and dragonflies over large distances (hundreds of kilometers) [7]. The researchers tagged the insects with 0.23 or 0.27 g commercial tags (Lotek NTQB-1) that emit OOK-modulated pings that can be individually identified. The insects were tracked using the Motus system [6]. Tracking individual insects remains a difficult challenge, especially over large distances.

The use ARGOS tags started with 180 g tags. The first prototype was deployed on a mute swan (*Cygnus olor*) in 1983, and the second tracked a bald eagle (*Haliaeetus leucocephalus*) for 9 months in 1984–1984. Similar tags were subsequently used to track golden eagles (*Aquila chrysaetos*) to aid in their conservation. By 1993, much lighter ARGOS tags weighing 27 g were available commercially and were used to track 50 peregrine falcons (*Falco peregrinus*) [27].

Wideband ToA tags were used to demonstrate that Egyptian fruit bats (*Rousettus aegyptiacus*) navigate using a cognitive map [5]. In this study, 172 bats carrying 4 g tags were tracked by the ATLAS system, yielding more than 18 million localizations. The same system was also used to track 99 much smaller Kuhl’s pipistrelle bats (*Pipistrellus kuhlii*) and to demonstrate that they too navigate using a cognitive map [4]. These tiny bats (mean body mass of 6 g) were tracked with 0.76 g tags. The discovery that bats that navigate both visually (the Egyptian fruit bats) and using echolocation (Kuhl’s pipistrelle) perform this complex task using a cognitive map is significant; both studies were published in *Science*.

## 10. Discussion

Wildlife tracking tags provide highly valuable data for both science and conservation. Many species require miniature highly specialized tracking tags, in many cases radio-transmitting tags. Designing and manufacturing these tags remains a difficult challenge, primarily because this is a niche market and because different species require different tags. The requirement differences encompass aspects like mass, attachment methods, radio range, sampling rate, and accuracy.

To address this challenge, miniature radio-transmitting tags used off-the-shelf components in innovative ways, often relying on unconventional engineering practices. The seminal Cochran and Lord tag [13] and its pinging variant [48] provide an excellent example (both variants are depicted in Figure 2). Benson explains that these designs, as well as other contemporary efforts, borrowed ideas from the design of the first American satellite radio beacons [84,85,86]. However, Cochran and his collaborators understood that producing long-range miniature tags requires unconventional circuit-design and circuit-construction techniques; contemporary efforts that relied on conventional design techniques failed [84]. A comparison of the Cochran–Lord design to the slightly earlier and much more conventional design by LeMunyan et al. [14] also helps clarify just how innovative the Cochran–Lord design was.

In particular, Cochran and his team broke a key engineering principle in order to achieve their goals: modularity. Their tags used a single transistor to serve as a multivibrator, an RF oscillator, and a power amplifier. Similarly, the same single-turn coil was used as a tank inductor and as an antenna. As components became smaller, modular designs became feasible.

Today, the design and manufacture of miniature ratio transmitters for wildlife tracking faces three other challenges:

1. Modern miniature radio transmitters utilize highly integrated system-on-a-chip (SOC) integrated circuits, especially RF microcontrollers. Commercial RF SOCs combine sets of features that are designed to support mass market applications, such as Bluetooth gadgets, smart home appliances, utility meters, etc. These sets of features are not optimal for wildlife tracking tags. For example, many RF SOCs now combine power management subsystems like buck or boost converters, but not nano-power energy-harvesting converters. Another example is the resonator: while it is technically feasible to include a crystal or acoustic resonator in the same package as the RF SOC (as evidenced by the integrated clock chip used in [16] or the bulk acoustic wave resonator used in the CC2652RW 2.4 GHz RF microcontroller), most RF SOCs require an external clock that limits miniaturization. No current RF microcontroller supports high-data-rate BPSK, although this is clearly possible [32]. Finally, most RF microcontrollers come in 4-by-4 mm quad-flat no-leads (QFN) packages or larger, which are small enough for commercial applications, not in the much smaller wafer-level chip-scale packaging (WLCSP) packages (e.g., 1.4 by 1.5 mm).
2. Some specialized components are not mass produced and are hence not available, probably due to a too-small market. For example, thin gold-coated steel wire would be ideal for antennas and can be manufactured, but it is essentially impossible to source commercially.
3. Spectrum sharing with other users. In much of the world, there is no specific frequency allocation for wildlife tracking [87], so most tags transmit in license-free industrial, scientific, medical (ISM) bands or in license-free bands where non-specific short-range devices (SRD) are allowed (the term non-specific means that the devices do not fall under some application-specific regulation, such as regulation of remote metering or wireless alarm devices). European regulation states that non-specific SRD devices transmitting up to 10 mW with a duty cycle of at most 10% may transmit in the 433.05–434.79 MHz band with no bandwidth limit [88] (Annex B, Line H). Almost all countries allow license-free transmission in this band. Some other SRD and ISM bands are restricted to specific regions or countries. For example, the same European regulation allows non-specific SRD devices in the 865–868 MHz band, but license-free transmission on this band is prohibited in the US. License-free bands are often heavily congested, leading to interference and inconsistent tracking performance.

These challenges, especially the first two, mainly limit the ability to track very small species, including many small birds, bats, rodents, and insects. On large animals, ones that can carry tags of around 2.5 g ore more, current tags perform well. Sub-1 g tags do exist [12,16,35], but their life spans and capabilities are much more limited, especially at or below 0.5 g. One exception that seems to be emerging is BLE tags, which can be miniaturized well because highly integrated chips with precisely the correct mix of features for them do exist.

## Figures and Tables

**Table 1:** Properties of wildlife tracking transmitters from the viewpoint of users.

Property	Significance to Users
Mass and size	Smaller tags can track smaller animals; tags heavier than some fraction of body mass cannot be used due to the risk that they might perturb the behavior of the animal and might threaten its survival or ability to raise offspring.
Antenna	The antenna should not pose a risk of entanglement. Short whips do not; loops do. The antenna should also not cause too much drag on flying animals. It should not be possible for the animals or conspecifics to damage or remove it (e.g., by pulling with a beak, chewing, etc.).
Effective life span	The longer the tag transmits, the longer the animal can be tracked. Tracking duration affects which scientific questions can be investigated by the tracking and which cannot.
Temporal resolution	Frequent transmissions tend to generate more localizations, allowing movement tracks to be estimated more accurately. Frequent transmissions shorten life spans when primary batteries are used, a common case.
Compatibility with receiving systems	Transmitting tags should be compatible with the receivers and the localization system that are supposed to track them. Some tags are compatible with multiple localization systems and receivers, some are specialized to a particular system.
Activation mechanism	Tags whose transmission can be turned in the field just prior or just after the tag is attached to an animal do not waste some of their life span before they are actually attached to an animal.
Sensors and data storage/upload	Sensors that measure the environment of the animal or its behavior and physiology provide valuable data for some investigations. Sensor data are sometimes stored on the tag (which must be retrieved to download the data) and sometimes transmitted wirelessly. Transmitting data tend to use a lot of energy; storing it less so.
Cost	Inexpensive tags enable tagging of more individual animals with a given research budget, providing stronger statistical evidence.
